# Contralateral cerebello-thalamo-cortical pathways with prominent involvement of associative areas in humans in vivo

**DOI:** 10.1007/s00429-014-0861-2

**Published:** 2014-08-19

**Authors:** Fulvia Palesi, Jacques-Donald Tournier, Fernando Calamante, Nils Muhlert, Gloria Castellazzi, Declan Chard, Egidio D’Angelo, Claudia A. M. Wheeler-Kingshott

**Affiliations:** 1 Department of Physics, University of Pavia, Via Bassi 6, 27100 Pavia, Italy; 2 Brain Connectivity Center, C. Mondino National Neurological Institute, Via Mondino 2, 27100 Pavia, Italy; 3 The Florey Institute of Neuroscience and Mental Health, Melbourne Brain Centre, 245 Burgundy Street, Heidelberg, VIC 3084 Australia; 4 Department of Medicine, Austin Health and Northern Health, University of Melbourne, Studley Road, Heidelberg, Australia; 5 Department of Neuroinflammation, NMR Research Unit, Queen Square MS Centre, UCL Institute of Neurology, Queen Square, London, WC1N 3BG UK; 6 Department of Psychology, Cardiff University, Cardiff, CF10 2AT UK; 7 Department of Industrial and Information Engineering, University of Pavia, Via Ferrata 1, 27100 Pavia, Italy; 8 Department of Brain and Behavioural Sciences, University of Pavia, Via Forlanini 6, 27100 Pavia, Italy; 9 National Institute for Health Research, University College London Hospitals Biomedical Research Centre, 149 Tottenham Court Road, London, W1T 7DN UK

**Keywords:** Cerebral cortex, Cerebellum, Diffusion MRI, MRI tractography

## Abstract

In addition to motor functions, it has become clear that in humans the cerebellum plays a significant role in cognition too, through connections with associative areas in the cerebral cortex. Classical anatomy indicates that neo-cerebellar regions are connected with the contralateral cerebral cortex through the dentate nucleus, superior cerebellar peduncle, red nucleus and ventrolateral anterior nucleus of the thalamus. The anatomical existence of these connections has been demonstrated using virus retrograde transport techniques in monkeys and rats ex vivo. In this study, using advanced diffusion MRI tractography we show that it is possible to calculate streamlines to reconstruct the pathway connecting the cerebellar cortex with contralateral cerebral cortex in humans in vivo. Corresponding areas of the cerebellar and cerebral cortex encompassed similar proportion (about 80 %) of the tract, suggesting that the majority of streamlines passing through the superior cerebellar peduncle connect the cerebellar hemispheres through the ventrolateral thalamus with contralateral associative areas. This result demonstrates that this kind of tractography is a useful tool to map connections between the cerebellum and the cerebral cortex and moreover could be used to support specific theories about the abnormal communication along these pathways in cognitive dysfunctions in pathologies ranging from dyslexia to autism.

**Electronic supplementary material:**

The online version of this article (doi:10.1007/s00429-014-0861-2) contains supplementary material, which is available to authorized users.

## Introduction

The cerebellum is a brain structure forming complex large-scale connections, whose integrative functions are still poorly understood. Besides a well-known role in motor learning and control (Holmes 1939; Evarts and Thach 1969), recent works have demonstrated a crucial role of the cerebellum in a number of other functions including cognition (Middleton and Strick 1994; Schmahmann and Caplan 2006). Tract-tracing and functional investigations both in non-human primates and in humans have shown projections from the dentate nucleus of the cerebellum to prefrontal and posterior parietal cortices via the thalamus supporting the hypothesis of a significant role for the cerebellum in higher cognitive and emotional processes (Middleton and Strick 1994; Schmahmann and Pandya 1995; Kelly and Strick 2003; Ramnani 2006; Strick et al. 2009). However, evidence in humans is more limited compared to that in non-human primates due to technical challenges of assessing in vivo the long polysynaptic connections between the cerebellum and the cerebral cortex (Snider and Eldred 1952; Nandi et al. 2002).

Recent developments in MRI technology have enabled the study of the anatomical cerebellar connections in vivo in humans using diffusion tensor imaging (DTI) and tractography (Habas and Cabanis 2007a, b; Jissendi et al. 2008; Doron et al. 2010; Anderson et al. 2011; Hyam et al. 2012). These techniques have already provided a visualization of afferent and efferent projections through the superior cerebellar peduncles (SCPs), the red nuclei (RN) and the thalamic projections to the cortex (Behrens et al. 2003a; Salamon et al. 2005). A major problem of these studies is that the diffusion tensor model has intrinsic limitations; in particular, it does not directly resolve crossing fibre structures (Alexander et al. 2001, 2002; Tuch et al. 2002; Jissendi et al. 2008; Tournier et al. 2011). The consequence is that tractography methods based on the diffusion tensor (DT) properties allow only partial reconstruction of cerebellar white matter tracts, and therefore have limited capability to reveal complex anatomical cerebello-thalamo-cortical circuits (Salamon et al. 2007). Some investigations have used alternative techniques, which overcome the intrinsic limitations of the DT model: diffusion spectrum imaging (DSI) (Wedeen et al. 2005) was used to study the intra-cerebellar connections in vivo in humans (Granziera et al. 2009) while multi-tensor reconstruction (Behrens et al. 2007) and constrained spherical deconvolution (CSD) (Tournier et al. 2007, 2012) were used to identify the dentate-rubro-thalamic pathway, originating from the dentate nucleus in the cerebellum and terminating in the contralateral ventrolateral (VL) and ventroanterior (VA) nuclei of the thalamus (Kwon et al. 2011; Van Baarsen et al. 2013; Akhlaghi et al. 2013). However, nobody has yet reconstructed the cerebello-thalamo-cortical pathway respecting the predicted decussation occurring just after the exit of the pathway from the SCP and leading it to the contralateral thalamus and cerebral cortex in a cohort of healthy subjects.

**Fig. 1:**
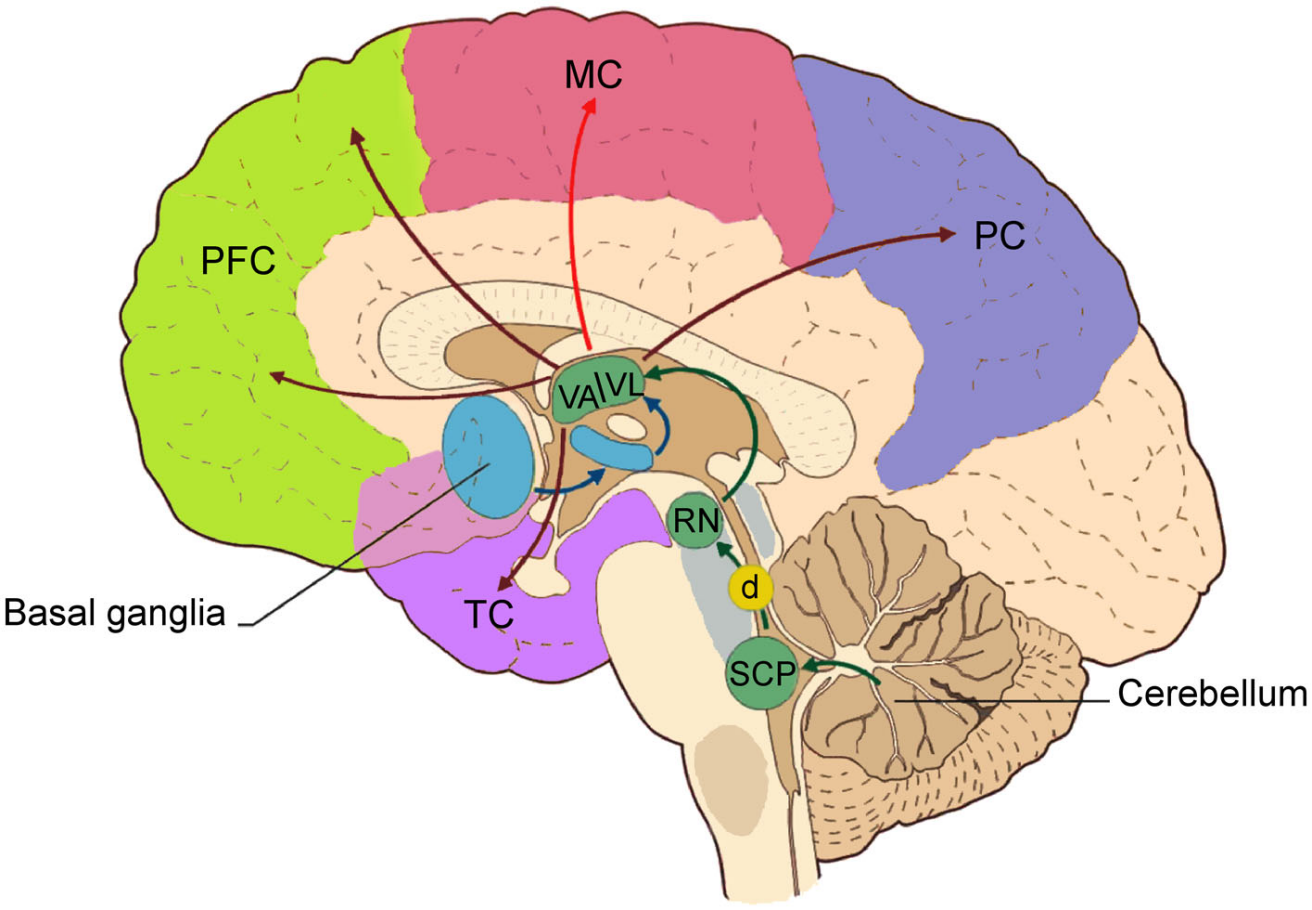
The most important connections in the cerebello-cortical circuit. Projections from the basal ganglia (through the subthalamic nucleus, STN) go mainly to the thalamic nuclei (VA/VL). The cerebellum sends its output through the superior cerebellar peduncle (SCP), the contralateral red nucleus (RN), and VA/VL of the thalamus to various cerebral areas including the motor cortex (MC), the prefrontal cortex (PFC), the parietal cortex (PC), and the temporal cortex (TC). The decussation (d) of the cerebello-thalamo-cortical pathway is indicated by the *yellow circle*. Modified from D’Angelo and Casali (2013)

In this paper, we used advanced diffusion imaging methods to reconstruct, in humans in vivo, the pathway connecting the cerebellar cortex to the contralateral cerebral cortex, passing through the SCP, the RN and the thalamus. Figure 1 shows a schematic view of the most important connections that we expect to find in the cerebello-cerebral circuit. While recognizing that tractography provides only indirect evidence of anatomical connectivity between regions and cannot distinguish between direct connections and pathways involving synapses (like the cerebello-thalamo-cortical pathway) (Catani et al. 2012; Jones et al. 2013), we aimed to assess the usefulness of tractography for investigations of such large-scale neural circuits. In particular, we aimed to ascertain whether (1) pathways connecting the cerebellar cortex with the contralateral cerebral cortex can be reconstructed from in vivo diffusion data; (2) there is a consistency of the tract involvement in cortical areas of the cerebrum and cerebellum with similar function or anatomical meaning; (3) the majority of streamlines passing through the SCP connects the cerebellar hemisphere with contralateral associative areas, as has been hypothesized based on the supposed parallel evolution of these two brain structures (Sultan 2002). Achieving these aims would support the hypothesis that the cerebellum takes part in central circuits involved in higher brain functions and cognitive processing (Habas et al. 2009; Krienen and Buckner 2009; Buckner et al. 2011), and underpin future studies of abnormal communication along these pathways, which could be implicated in pathologies recently shown to involve the cerebellum, such as dyslexia and autism (Schmahmann and Caplan 2006; D’Angelo and Casali 2013).

## Materials and methods

In this paper, the reconstruction of the contralateral cerebello-thalamo-cortical pathway was achieved by combining two advanced diffusion techniques: tract reconstruction based on CSD, which can model multiple fibre populations within a voxel and is able to resolve the decussation of the trans-hemispheric connection, and super-resolution maps based on track-density imaging (TDI) (Calamante et al. 2010), which allowed accurate seed and target region placement. TDI maps improve resolution and white matter contrast compared with conventional DTI maps (such as mean diffusivity, MD, and fractional anisotropy, FA) and can be generated from high angular resolution diffusion imaging (HARDI) datasets (Calamante et al. 2010). After reconstruction of the cerebello-thalamo-cortical connections, a number of “tractography metrics” were defined in an attempt to quantify the pattern of the connections for specific cerebral and cerebellar cortical regions. Given the well-known challenges in quantifying connectivity based on tractography (Jones et al. 2013), we defined two simple metrics that, while imperfect, should nonetheless provide sufficiently robust evidence to support our conclusions. These are the proportion of each cortical region that is reached by the tractography algorithm and the proportion of the total cortical volume reached by the tractography algorithm that is contained within each cortical region; neither of these is expected to be overly influenced by streamline count. For completeness, we also report the streamline counts reaching each cortical region.

### Subjects

The study was carried out on 15 right-handed healthy adults (7 males and 8 females; mean age 36.1 years and range 22–64 years) with no previous history of neurological symptoms. All participants gave written informed consent. The study protocol was approved by the local institutional research ethics committee.

### MRI acquisition

All data were acquired on a Philips Achieva 3T MRI scanner (Philips Healthcare, Best, The Netherlands) using a 32-channel head coil. The HARDI scan consisted of a cardiac-gated SE echo-planar imaging (EPI) sequence acquired axial-oblique and aligned with the anterior commissure/posterior commissure line, for a total scan time of approximately 20 min. The imaging parameters were TR ≈ 24 s (depending on the cardiac rate), TE = 68 ms, SENSE factor = 3.1, acquisition matrix = 96 × 112, 2 mm isotropic voxel and 72 axial slices with no gap. The diffusion weighting was distributed along 61 optimized non-collinear directions with a *b* value of 1,200 s/mm^2^ (Cook et al. 2007). For each set of diffusion-weighted data, 7 volumes with no diffusion weighting (b0) were acquired. For anatomical reference a whole brain high-resolution 3D sagittal T1-weighted (3DT1w) fast field echo (FFE) scan was acquired using the following parameters: TR = 6.9 ms, TE = 3.1 ms, TI = 824 ms, acquisition matrix = 256 × 256, 1 mm isotropic voxel, 180 sagittal slices, acquisition time 6 min 31 s.

### Diffusion analysis and fibre tracking

HARDI data were analysed using the FSL (FMRIB Software Library, http://fsl.fmrib.ox.ac.uk/fsl/fslwiki/) and MRtrix (http://www.brain.org.au/software/mrtrix/) software packages, following these steps:

1. Pre-processing: Eddy current correction and brain extraction (Smith 2006) were performed using FSL.
2. Structural-diffusion data alignment: The high-resolution 3DT1w volume was realigned to the diffusion data by inverting the full-affine transformation (12 degrees of freedom, FLIRT, FSL) (Jenkinson et al. 2002) from diffusion to high-resolution space.
3. Decussation realignment: For each participant, the 3DT1w volume in diffusion space (obtained in step 2) was realigned along the superior/inferior direction to the MNI-152 template using a rigid body transformation (6 degrees of freedom) with nearest neighbour interpolation. This transformation was chosen to align the decussation region between all subjects to compare parameter values along the aligned tracts while minimizing potential biases that could be introduced when using non-linear registration of diffusion data; hence, we chose to perform the analysis in the individual subjects’ space. The transformations were then applied to the diffusion-weighted data. This space will be considered the subject’s native space from this point onward, rather than the acquired space.
4. Whole brain tractography: To generate the TDI maps, whole brain tractography was performed with MRtrix using an algorithm that combines the CSD technique with probabilistic streamlines tractography (Tournier et al. 2012); the relevant parameters were seed = whole brain, step size = 0.1 mm, maximum angle between steps = 10°, maximum harmonics order = 8, termination criteria: exit the brain or when the CSD fibre-orientation distribution amplitude was <0.1. Streamlines were generated by randomly seeding throughout the whole brain until the desired total of 2.5 million streamlines had been selected.
5. TDI map: From the streamlines obtained in step 4, a TDI map was created as the total number of streamlines passing within each element of a user-defined super-resolution grid (Calamante et al. 2010); for this study a 1-mm resolution grid was used.
6. Cerebello-thalamo-cortical pathways: Cerebello-thalamo-cortical pathways were reconstructed by combining the CSD algorithm with probabilistic tractography and by tracking the bundle passing through two regions of interest (ROIs) (Schmahmann et al. 1999; Habas and Cabanis 2007a, b; Kwon et al. 2011): the SCP and the contralateral RN. These pathways were reconstructed by randomly seeding streamlines throughout the SCP seed ROI (see step 7) until 3,000 streamlines were reconstructed. For clarity, from this point onward the word “tract” is meant to indicate the tractography reconstruction of the cerebello-thalamo-cortical connection. To compare this with the conventional diffusion tensor model, cerebello-thalamo-cortical pathways were also reconstructed using a DTI-based streamline deterministic tractography by randomly seeding streamlines throughout the seed ROI defined by the SCP, and using the following parameters: step size = 0.1 mm, maximum angle between steps = 4.5°, initial FA ≥0.2, termination criteria: exit the brain or when the FA was <0.1; once again, a total of 3,000 streamlines were reconstructed. No contralateral target ROI was defined because with this approach tracts run only ipsilaterally.
7. Seed/target ROIs placement: SCP and RN masks were placed using the high-resolution TDI images. The seed ROI was defined as a sphere with 2 mm radius centred on the SCP in each cerebellar hemisphere and was identified in the coronal plane, as described by Calamante et al. (2010), while the target ROI on the whole contralateral RN was recognized as a very hypointense region (Calamante et al. 2013).
8. MNI normalization: 3DT1w images from all participants were normalized to the MNI-152 template using a non-linear registration algorithm with nearest neighbour interpolation from the FSL library (FNIRT) (Klein et al. 2009).
9. Atlases-diffusion data alignment: The atlas of Brodmann areas (BA) and of the cerebellum (SUIT) (Diedrichsen et al. 2009) was aligned to native space of each subject by inverting the warping transformation obtained in step 8 to more accurately study the cerebellar pathways.
10. Parcellation of cerebral and cerebellar cortices: For all participants, in native space, cerebral and cerebellar cortices were parcellated in two ways: one based on anatomical grounds, the other on a functional basis.
  Anatomical parcellation consisted of the following areas:
  Cerebrum: prefrontal cortex, frontal, parietal, temporal, occipital and limbic lobes (Brodmann 2006);
  Cerebellum: anterior, VI, lateral Crus I–II, VIIb/VIII and inferior lobules (Schmahmann et al. 1999).
  Functional parcellation consisted in the following areas:
  Cerebrum: motor, associative, primary sensory, primary auditory and primary visual areas (Brodmann 2006);
  Cerebellum: primary motor, sensory motor and cognitive/sensory areas (Diedrichsen et al. 2009).
  Deep grey matter nuclei were segmented using FIRST (FSL). The following areas were considered in the analysis: basal ganglia (caudate, putamen and pallidus), thalamus, nucleus accumbens, amygdala and hippocampus.
11. Quantification of cROI_tract_, i.e. the percentage of each cortical region (cROI) within the tract: as shown by Fig. 2 this index reflects the proportion of the parcellated cortical region under consideration (cROI) that is involved in the cerebello-thalamo-cortical pathway. For each parcellation and for each subject in native space, cROI_tract_ was calculated as the percentage number of voxels within the parcellation that were reached by any number of streamlines within the tract.

**Fig. 2:**
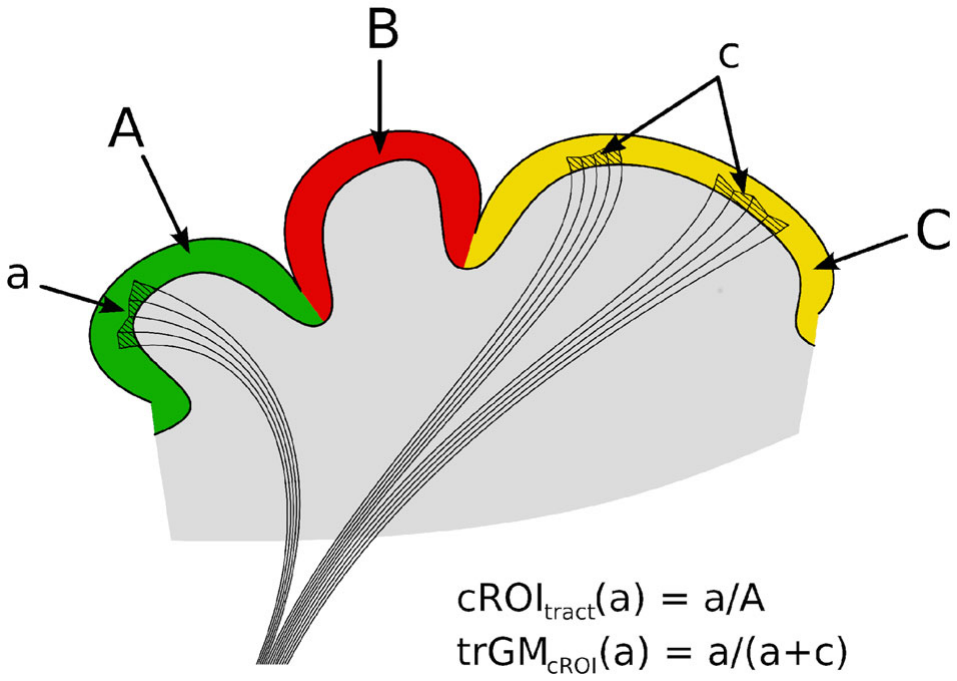
The two tractography metrics: cROI_tr_ and trGM_cROI_. Each *different colour* (*green*, *red*, *yellow*) and *capital letter* represents a different cortical parcellation (cROI) while each *lowercase letter* represents the cortical region reached by the tract for each cROI
12. Quantification of trGM_cROI_, i.e. the proportion of the overall tract grey matter (GM) belonging to a specific cROI: as shown by Fig. 2 this index reflects the proportion of the total cortical GM involved in the tract that belongs to a particular parcellated cortical region. For each parcellation and for each subject in native space, trGM_ROI_ was calculated as the percentage of all cortical voxels reached by any streamline in the tract that belong to the particular cROI of interest. This analysis was also performed for the deep GM nuclei, by computing the metric over all voxels within the deep GM regions.
13. Quantification of TSC, i.e. the total streamline count: this measure reflects the number of the streamlines reaching the cortex rather than the number of voxels of the tract included in the cortex. For each cortical parcellation TSC was calculated with MRtrix isolating from a tract only the streamlines that entered a given region.
14. Mean cerebello-thalamo-cortical pathway: To assess the consistency of the tracts in MNI space and for display purposes the tracts from all subjects were normalized using the same transformation calculated for 3DT1w images in step 8. A mean image of tracts was calculated from the binarized tracts for each subject (Ciccarelli et al. 2003). Voxels were assigned the count of the number of subjects with that specific voxel included in the mask. The mean tracts’ image was thresholded to include voxels common to at least 20 % of subjects. The unthresholded left and right mean tracts in MNI space are available on request.
15. Thalamus parcellation: Since the thalamus is a synaptic relay, to assess whether the reconstructed cerebello-thalamo-cortical pathways actually reflected the thalamo-cortical connectivity, the thalamus was parcellated as indicated by Behrens et al. (2003a, b) and the mean cerebello-thalamo-cortical pathway was superimposed in MNI space.

## Results

### The reconstruction of the cerebello-thalamo-cortical pathway

**Fig. 3:**
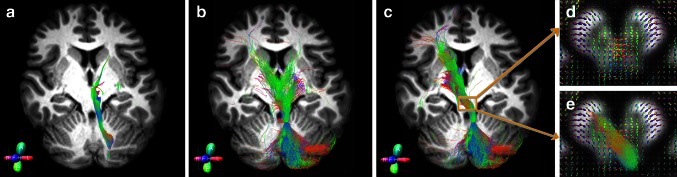
Example of cerebello-thalamo-cortical pathway from a representative subject. This is a 2D rendering of streamlines extending over a volume of 5 mm, but mapped to a section of 1 mm thick. The same seed ROI (**a**–**c**) was placed on left superior cerebellar peduncle. **a** The tract was reconstructed using DTI and streamline tractography. No target ROI was drawn. **b** The tract was reconstructed using a combination of the CSD algorithm and probabilistic tractography. No target ROI was drawn. **c** The tract was reconstructed as in **b** with a target ROI drawn on the whole contralateral red nucleus. **d** Details of the fibre-orientation distribution (FOD) within the decussation region. **e** Details of the FOD and tract within the decussation region

**Fig. 4:**
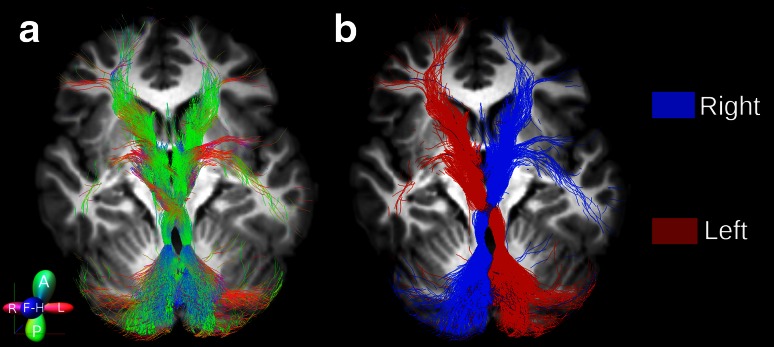
2D rendering of both left and right cerebello-thalamo-cortical pathways from a representative subject. **a** The tracts are *colour*-coded by direction to follow the anatomy and the directionality of both left and right tracts. **b** The tracts are reported using a single *solid colour* for each tract to distinguish the streamlines from the left (*red*) and right (*blue*) pathways

The combination of using the CSD algorithm and probabilistic tractography successfully reconstructed the cerebello-thalamo-cortical pathways in all subjects. Seeding from the SCP, streamlines were identified connecting the cerebellar cortex to the contralateral cortical hemisphere, passing through the contralateral RN. Figure 3 shows a comparison between the cerebello-thalamo-cortical pathway reconstructed using DTI and the streamline deterministic tractography (Fig. 3a) and a combination of CSD and the probabilistic tractography (Fig. 3b) in a representative subject. As can be seen in Fig. 3a, the DTI approach fails to reconstruct contralateral connections, which is a problem that cannot be resolved even with the usage of a contralateral target ROI. To select the contralateral connections it is therefore necessary to start from a non-tensor-based approach such as CSD, which produces streamlines running both ipsi and contralaterally (Fig. 3b); to isolate just the contralateral pathway it is necessary to add of a target region that we chose to be the contralateral red nucleus (Fig. 3c). Figure 4 also shows a 2D rendering of both cerebello-thalamo-cortical pathways from a representative subject. In particular, Fig. 4a shows the tracts colour-coded by direction in order to represent their anatomy, while Fig. 4b shows the same tracts using a single colour per tract in order to distinguish left from right-side streamlines highlighting their extensions into the cerebral and cerebellar cortices.

**Fig. 5:**
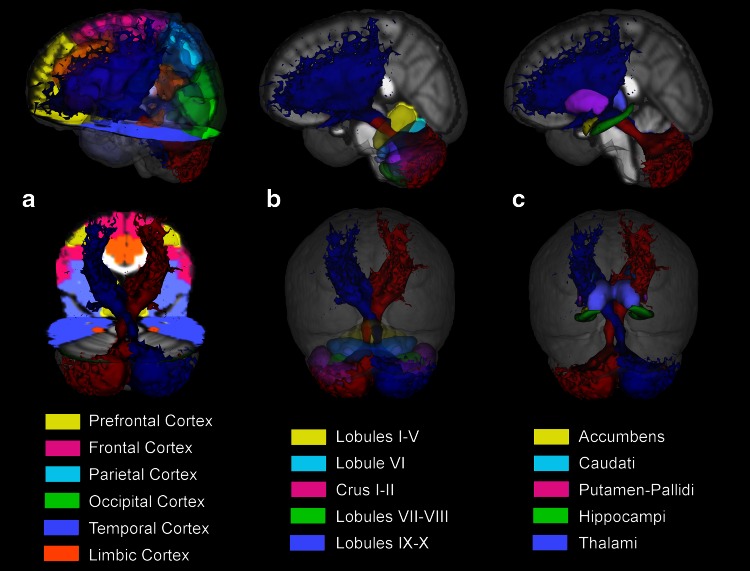
Tridimensional view of the average cerebello-thalamo-cortical pathway across all subjects in MNI space. Cerebral (**a**), cerebellar (**b**) and deep grey matter (**c**) atlases are overlaid to assist visualization of cerebellar connections. **a** Distribution of left (*red*) and right (*blue*) tracts in the cerebral cortex: the reconstructed tracts reach the prefrontal (*yellow*), frontal (*fuchsia*) and temporal (*violet*) cortices with greater density of streamlines. **b** Streamlines distribution in the cerebellar cortex: the lateral Crus I–II (*fuchsia*) and the lateral lobules VIIb/VIII (*green*) are showing the greatest density of tracts. **c** Streamlines distribution of deep grey matter nuclei: the thalami (*violet*), the caudate (*light blue*) and the putamen (*fuchsia*) show the greatest trGM_cROI_

To highlight the extent of the cerebello-thalamo-cortical pathway, Fig. 5 shows different views of the average tract across all subjects in MNI space. Figure 5a shows the distribution of streamlines in the cerebral cortex: the reconstructed tracts reach the prefrontal, frontal and temporal cortices with a high density of streamlines. Figure 5b shows streamlines distribution in the cerebellar cortex: the highest density of streamlines is observed in lateral Crus I–II and in lateral lobules VIIb/VIII. Figure 5c shows that specific deep grey matter nuclei were reached by a high number of streamlines, especially the VA and VL nuclei in the thalami and the caudate nuclei (but also putamen and pallidus) in the basal ganglia. The tracts also show that most of the cerebellar streamlines are ipsilateral to the SCP seed, with a minimal portion of streamlines crossing contralaterally; the connection to the cortex, instead, runs contralaterally to the SCP seed (as imposed by the presence of the waypoint region of the contralateral RN) with a small number of streamlines running into the septum.

For completeness, we have also displayed the cerebello-thalamo-cortical pathway from cerebellar cortex to cerebral cortex by showing slice-by-slice its extension in Supplementary Materials (Supplementary Figure 1). This also demonstrates connections between the cerebellum and septal regions. For simplicity, we have chosen to show only the cerebello-thalamo-cortical pathway seeded in the left SCP.

**Fig. 6:**
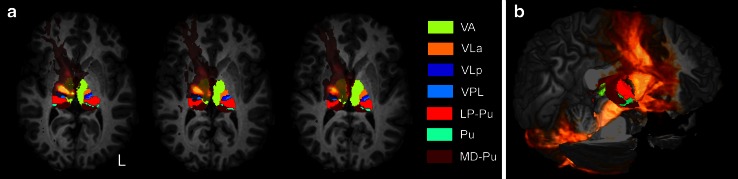
Extension of the left cerebello-thalamo-cortical pathway overlapped to the parcellated thalami in a representative subject. *L* indicates the left side of the brain. **a** 2D rendering: the highest density of streamlines is seen in the VA and VL nuclei of the thalamus, which correspond to areas principally connected with prefrontal (*yellow*) and frontal (*orange* and *blue*) cortices. **b** Tridimensional representation of the tract: the VA and VL nuclei of the right thalamus (*yellow*, *orange* and *blue*) are hidden from the tract

To highlight the extension of the tract in the thalamic relay, Fig. 6 shows different views of the average tract across all subjects overlaid onto the parcellated thalamus in MNI space. The highest density of streamlines is seen in the VA and VL nuclei of the thalamus, which correspond to areas principally connected with prefrontal and frontal (motor) cortices (Behrens et al. 2003a; Zhang et al. 2008; Mang et al. 2012). A few streamlines reached the anterior thalamic area and from there the temporal lobe. An even smaller contingent of streamlines reached posterior thalamic nuclei and the pulvinar and from there the parietal and occipital lobes.

### Tractography metric results

The destination of SCP streamlines in different brain structures, including cerebellar and cerebral cortices and deep grey matter nuclei, was evaluated using three parameters: cROI_tract_, trGM_cROI_ and TSC (see steps 11–13 of the “Materials and methods”). In turn, cerebral and cerebellar cortices were parcellated into two sets of regions based on either their anatomical or functional basis (see step 10 of the methods) (Schmahmann et al. 1999; Diedrichsen et al. 2009).

*Anatomical parcellation* Table 1 reports cROI_tract_, trGM_cROI_ and TSC (averaged across subjects) for left and right tracts added together in all cerebral and cerebellar cortical areas as defined by anatomical parcellation. In the cerebrum, the prefrontal cortex showed the highest value of the three tractography metrics. In the cerebellum, the area of lobule VIIb–VIII showed the highest value of cROI_tract_ and Crus I–II showed the highest value of trGM_cROI_, while the anterior lobule showed the highest TSC value.

**Table 1:** ROI_tract_, trGM_cROI_ and TSC values in cerebral and cerebellar cortical areas defined on anatomical bases

Structure	cROI_tract_ Mean (SD) (%)	trGM_cROI_ Mean (SD) (%)	TSC Mean (SD)
Cerebrum			
Prefrontal cortex	**5.6 (1.4)**	**38 (11)**	**2,013 (478)**
Frontal lobe	2.9 (1.2)	16 (5)	736 (267)
Parietal lobe	0.7 (0.3)	4 (2)	169 (91)
Temporal lobe	2.5 (0.7)	35 (5)	1,778 (441)
Occipital lobe	0.6 (0.7)	3 (3)	290 (171)
Limbic lobe	1.4 (0.5)	3 (1)	233 (85)
Cerebellum			
Anterior lobule (I–V)	4.9 (1.3)	4 (1)	**5,192 (867)**
Lobule VI	9.3 (3.1)	10 (3)	1,202 (525)
Lateral Crus I–II	19.9 (2.6)	**48 (4)**	2,861 (469)
Lobules VIIb/VIII	**20.4 (4.2)**	31 (5)	3,024 (634)
Inferior lobule (IX–X)	9.6 (3.5)	5 (3)	1,805 (874)

Data are expressed as mean (SD) for each brain area. Left and right measurers were added together. Bold values represent the maxima values

*cROI*_*tract*_ percentage of each cortical region within the tract, *trGM*_*cROI*_ proportion of the overall tract grey matter belonging to a specific cortical region, *TSC* total streamline count

*Functional parcellation* Table 2 reports cROI_tract_, trGM_cROI_ and TSC (averaged across subjects) for left and right tracts added together in all cerebral and cerebellar cortical areas defined on their functional bases. In the cerebrum, the motor area showed the highest value of cROI_tract_, while the associative area showed the highest value of trGM_cROI_ and TSC. In the cerebellum, the sensory motor area showed the highest value of cROI_tract_, while the cognitive and sensory area showed the highest value of trGM_cROI_ and the primary motor area showed the highest value of TSC.

**Table 2:** ROI_tract_, trGM_cROI_ and TSC values in cerebral and cerebellar cortical areas defined on functional bases

Structure	cROI_tract_ Mean (SD)	trGM_cROI_ Mean (SD) (%)	TSC Mean (SD)
Cerebrum			
Motor area	**3.2 (1.6)**	14 (5)	608 (265)
Associative areas	2.8 (0.4)	**80 (8)**	**3,284 (336)**
Primary somatosensory area	1.6 (0.8)	2 (1)	111 (64)
Primary visual area	0.7 (0.6)	3 (3)	290 (171)
Primary auditory area	0.4 (0.4)	1 (1)	15 (10)
Cerebellum			
Primary motor area	4.9 (1.3)	4 (1)	**5,192 (867)**
Cognitive/sensory area	15.2 (1.9)	**79 (4)**	4,429 (365)
Sensory motor area	**58.1 (12.4)**	17 (3)	2,543 (688)

Data are expressed as mean (SD) for each brain area. Left and right measurers were added together. Bold values represent the maxima values

*cROI*_*tract*_ percentage of each cortical region within the tract, *trGM*_*cROI*_ proportion of the overall tract grey matter belonging to a specific cortical region, *TSC* total streamline count

*Deep grey matter parcellation* Table 3 reports cROI_tract_, trGM_cROI_ and TSC (averaged across subjects) for left and right tracts added together in deep grey matter nuclei. The pallidi showed the highest value of cROI_tract_, while the thalami showed the highest value of trGM_cROI_ and of TSC.

**Table 3:** cROI_tract_, trGM_cROI_ and TSC values in deep grey matter nuclei

Deep GM structure	cROI_tract_ Mean (SD)	trGM_cROI_ Mean (SD) (%)	TSC Mean (SD)
Caudati	32.7 (8.7)	26 (6)	1,440 (467)
Thalami	18.0 (6.2)	**34 (11)**	**3,491 (726)**
Accumbens	19.1 (10.1)	3 (2)	546 (528)
Amygdalae	1.0 (0.6)	0 (0)	37 (21)
Hippocampi	0.3 (0.2)	0 (0)	53 (124)
Pallidi	**39.4 (9.1)**	19 (4)	2,156 (705)
Putamen	13.5 (3.8)	18 (4)	1,552 (365)

Data are expressed as mean (SD) for each brain area. Left and right measurers were added together. Bold values represent the maxima values

*cROI*_*tract*_ percentage of each cortical region within the tract, *trGM*_*cROI*_ proportion of the overall tract grey matter belonging to a specific cortical region, *TSC* total streamline count

**Fig. 7:**
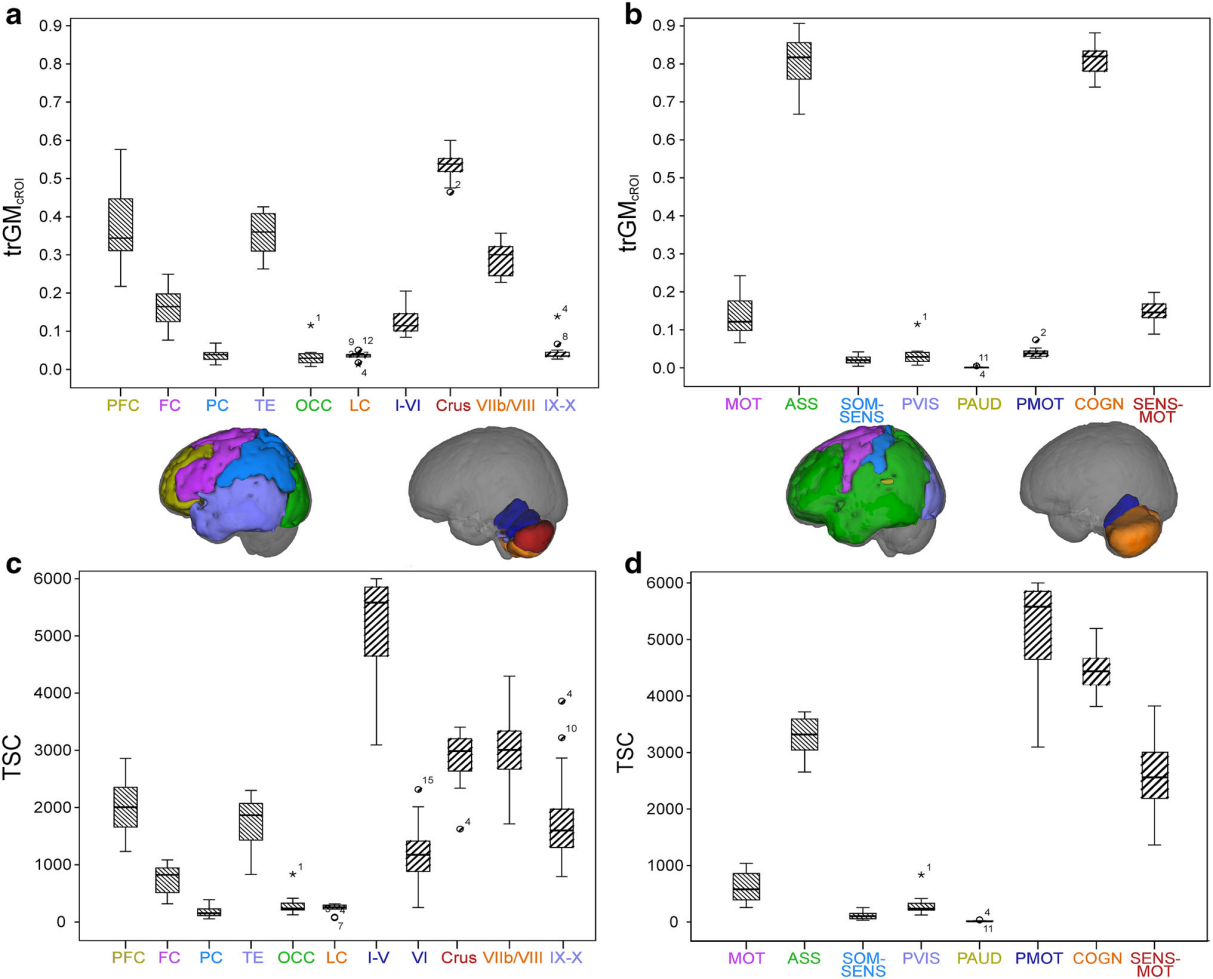
Histograms of mean values of trGM_cROI_ and TSC for each region of the cerebral cortex and of the cerebellar cortex. *PFC* prefrontal cortex, *FC* frontal cortex, *PC* parietal cortex, *TE* temporal cortex, *OCC* occipital cortex, *LC* limbic cortex, *MOT* motor, *ASS* associative, *SOM-SEN* somatosensory, *PVIS* primary visual, *PAUD* primary auditory, *PMOT* primary motor, *COGN* cognitive/sensory, *SEN-MOT* sensory motor. **a** trGM_cROI_ values of all cerebral and cerebellar regions created on anatomical bases. PFC and Lateral Crus I–II have the highest values in cerebrum and cerebellum, respectively. **b** TSC values of all regions created on anatomical bases. PFC and lobules I–V have the highest values in cerebrum and cerebellum, respectively. **c** trGM_cROI_ values of all regions created on functional bases. ASS and COGN areas have the highest values in cerebrum and cerebellum, respectively. **d** TSC values of all regions created on functional bases. ASS and PMOT areas have the highest values in cerebrum and cerebellum, respectively

Tractography suggests that the cerebello-thalamo-cortical pathway spreads out to many different areas of the brain. We also compared the proportions of the tract that reached the cerebellar and cerebral cortices in anatomically and functionally corresponding areas, providing evidence for the presence of structural connectivity between these regions. The results are visualized in Fig. 7, where the mean values of trGM_cROI_ and of TSC are shown for each parcellation of the cerebral and cerebellar cortices.

### Anatomical parcellation

The main findings from Table 1 are as follows:

- Correspondence between trGM_cROI_ of the anterior cerebellum (lobules I–V and lobule VI) and the cerebral frontal lobe, with values of 14 % ± 4 % and 16 % ± 5 %, respectively.
- Correspondence between trGM_cROI_ of the prefrontal cortex and the lateral Crus I–II, with values of 38 % ± 11 % and 48 % ± 4 %, respectively.

### Functional parcellation

The main findings from Table 2 are as follows:

- The hemispheres of the cerebellum and the cortical associative areas have comparable trGM_cROI_ of 79 % ± 4 % and 80 % ± 8 %, respectively.
- The primary auditory and visual cortices show negligible values of trGM_cROI_ of 1 % ± 1 % and 3 % ± 3 %, respectively.

## Discussion

In this study, we show that tractography can realistically be used to map the pathway connecting the cerebellar hemispheres with the contralateral cerebral cortex passing through the SCP, RN and thalamus, despite the complications introduced by synapses and the decussation just after the SCP. Based on the more details of the TDI maps, the SCP was used as seed and the contralateral RN as target. The combination of a CSD algorithm with probabilistic tractography then allowed the reconstruction of the cerebellar connections towards several regions of the cerebral cortex. Quantitative analysis of tract projections passing through the SCP and RN showed that the cerebellar hemispheres on one side and the associative cerebral cortex on the other encompassed about 80 % of the tract. Our findings provide structural reconstruction in humans in vivo of the crossed-fibre pathways from cerebellar to cerebral cortex in accordance with predictions by ex vivo anatomical investigations (Voogd 2003; Standring 2008) and support the hypothesis of a prominent connectivity of lateral cerebellum to contralateral associative areas (Buckner et al. 2011).

It is however important to emphasize that tractography methods are inherently incapable of distinguishing between single neuron pathways and connections involving synapses, such as those assessed in this study. Nevertheless, tractography is currently the only in vivo in human method for investigating structural connectivity of specific systems and can be used to obtain reliable results, provided certain conditions are met; these are discussed in detail in the “Limitations of the present study” section below.

### The reconstruction of the cerebello-thalamo-cortical pathway

Recent studies have assessed cerebellar tract reconstruction using different types of tractography in vivo in human subjects (Salamon et al. 2007; Habas and Cabanis 2007a, b; Jissendi et al. 2008; Granziera et al. 2009; Doron et al. 2010; Anderson et al. 2011; Kwon et al. 2011; Hyam et al. 2012). Most of these studies have investigated cerebello-thalamo-cortical pathways (Salamon et al. 2007; Habas and Cabanis 2007a, b; Jissendi et al. 2008; Doron et al. 2010; Anderson et al. 2011) and a few others have reconstructed intra-cerebellar pathways (Granziera et al. 2009; Takahashi et al. 2013; Dell’Acqua et al. 2013). Several of these studies used the DT model and showed pathways passing through the SCPs and running ipsilaterally towards the cerebral cortex (Salamon et al. 2007; Habas and Cabanis 2007a, b; Jissendi et al. 2008; Doron et al. 2010; Anderson et al. 2011; Hyam et al. 2012). Some other studies instead exploited more complex models to reconstruct portions of the pathway or the intra-cerebellum connections in vivo (Granziera et al. 2009; Kwon et al. 2011; Van Baarsen et al. 2013; Akhlaghi et al. 2013) and post-mortem (Takahashi et al. 2013; Dell’Acqua et al. 2013). The decussation of the SCP is expected to occur from classical neuroanatomical descriptions (Voogd 2003; Standring 2008), but its reconstruction using MRI techniques in vivo in humans has only been achieved by a few studies using advanced diffusion approaches (Tuch et al. 2002; Tuch 2004; Wedeen et al. 2008; Tournier et al. 2012; Fernandez-Miranda et al. 2012; Van Baarsen et al. 2013; Akhlaghi et al. 2013). To the best of our knowledge, only two studies used advanced techniques, e.g. CSD and probabilistic tractography, to reconstruct the dentate–rubral and dentate–thalamic pathways in pathological conditions. Akhlaghi et al. (2013) demonstrated that the dentate-thalamo-cortical tracts of patients with Friedreich ataxia showed a decreased FA value and an increased MD value compared with controls, while Van Baarsen et al. (2013) demonstrated, in a single patient with cerebellar mutism, that changes in FA and MD values along the dentate-rubro-thalamic tract and its alterations might be the cause of the mutism. Both of these studies assessed how specific pathologies affected structural characteristics of the tracts of interest rather than investigating cerebellar involvement in cognitive processes, therefore offering complementary information to our findings. These observations confirm the importance of anatomical and functional studies of cerebellar connections in understanding pathologies.

In this paper we have shown contralateral connections between the cerebellum and the prefrontal, frontal and parietal cortices via the thalamus in humans in vivo, which we achieved by implementing a pipeline with two key points: the selection of a non-Gaussian diffusion model and the definition of a seed and a target ROI (Palesi et al. 2013). We chose to combine a method based on CSD with probabilistic tractography, because this approach has been shown to allow tracking through complex crossing fibre regions (Tournier et al. 2012; Akhlaghi et al. 2013). Using this approach we could reconstruct the contralateral cerebello-thalamo-cortical pathways originating from both left and right SCPs, which are completely missed using DT-based tractography methods. In fact, streamline DTI tractography techniques are unable to resolve the convergence of differently oriented tracts into the same area, as occurs in the white matter in the medullary core of the cerebellum. This intrinsic limitation could only be partially overcome using probabilistic tractography. To better represent the fibre structure, non-tensor models must be used that are known to address these fibre-crossing issues. The further use of a seed and contralateral target ROI, placed on high-resolution TDI images, warranted the selection of streamlines crossing at the decussation point as we expected from anatomical knowledge. Notice that the use of a seed and contralateral target ROIs cannot help resolving the lack of crossing streamlines when using the DT model.

The pathways that we generated show anatomical consistency between subjects, involving several areas of the cerebellar cortex, cerebral cortex and deep grey matter nuclei, passing through the VA and VL nuclei of the thalamus, caudate and putamen. In particular, most cerebellar streamlines are ipsilateral compared to the SCP seed, while a minimal proportion of streamlines cross over to the contralateral side. These findings are anatomically plausible (Watt and Mihailoff 1983; Noda et al. 1990).

### Tractography metric results

Having shown that the cerebello-thalamo-cortical pathway reconstructed by tractography was in accordance with findings from tract-tracing studies, as discussed above, we introduced metrics that reflect how different grey matter areas could be involved in the reconstructed tracts, allowing us to make further observations regarding the characteristics of the cerebello-thalamo-cortical pathway. In particular, comparable proportions of cortex were reached by the tract in anatomically and functionally corresponding areas of the cerebellar and cerebral cortices. Thus, areas expected to be connected from functional studies are also characterized by similar tractography metrics, in accordance with suggestions of strong links between the developments of corresponding regions (Sultan 2002).

From the anatomical parcellation point of view (Table 1), our results were in agreement with classical literature results by finding that the anterior cerebellum (lobules I–VI) and the cerebral frontal lobe were similarly involved in the tract (trGM_cROI_ was 14 and 16 %, respectively). These findings support a correspondence between these two areas, in line with the expected topography of primary motor and premotor areas (Snider and Eldred 1952; Grodd et al. 2001; Kelly and Strick 2003). Indeed, these regions are known to be reciprocally connected and to subserve motor and premotor functions (Schmahmann et al. 1999; Diedrichsen et al. 2009; Krienen and Buckner 2009).

From a cognitive point of view we would expect to find anatomical correspondence between prefrontal cortex and the lateral Crus I–II (Habas et al. 2009; Krienen and Buckner 2009). Our data show that indeed there is similar involvement between lateral Crus I–II and prefrontal areas (trGM_cROI_ was 48 ± 4 and 38 ± 11 %, respectively). Furthermore, our results are supported by recent tract-tracing and electro-physiological studies on primates and rats demonstrating that the cerebellum is effectively linked to the prefrontal cortex forming “closed-loop” connections (Middleton and Strick 2001; Mittleman et al. 2008; Arguello et al. 2012; Watson et al. 2014). Indeed, the cerebellar pathway extended considerably into prefrontal cortical areas in agreement with ex vivo anatomical determinations, which have shown that the cerebellum is reciprocally connected with the medial prefrontal cortex (PFC) (Watson et al. 2009), the dorsolateral PFC (Kelly and Strick 2003), and the anterior PFC (Krienen and Buckner 2009). The medial PFC is important in saccadic movements and cognitive control (Ridderinkhof et al. 2004) and is strongly involved in determining behaviour on the basis of expectations (Amodio and Frith 2006). Moreover, this cortical area plays a key role in fear extinction processes (Morgan et al. 1993; Milad and Quirk 2002). The dorsolateral PFC is particularly important in working memory (Petrides 2000), mental preparation for imminent actions (Pochon et al. 2001), and procedural learning (Pascual-Leone et al. 1996) and its functional alteration is involved in major psychoses (Weinberger et al. 1986, 1988; Dolan et al. 1993). The anterior PFC is less well understood (Ramnani and Owen 2004) but its main function could be that of integrating multiple distinct cognitive processes during goal-directed complex behaviours. Therefore, the fact that there is a possible correspondence of tractography metrics between cortices with similar functional roles, as reported here, supports the hypothesis of a route through which the cerebellum can influence both cognitive tasks through connections with various areas of the PFC and sensory and motor tasks through connections with frontal and parietal cortices (Schmahmann and Pandya 1993; D’Angelo and Casali 2013).

The observation that the parietal cortex only encompassed 4 % of the tract-connected GM is likely due to the low number of streamlines connecting between the cerebellum and the posterior thalamic nuclei (Fig. 6). Indeed studies focused on thalamic connectivity (Behrens et al. 2003b; Zhang et al. 2008; Mang et al. 2012) have demonstrated that the VA and VL nuclei of the thalamus are mainly connected with motor areas and the prefrontal cortex rather than the parietal cortex, which is in turn principally connected with the posterior thalamic nuclei receiving somatosensory information from pathway ascending from spinal cord and brain-stem. From ex vivo experiments it is known that the cerebellum sends outputs through the posterior VL of thalamus to the inferior parietal lobe (Clower et al. 2001), which is involved in response to the sight of an object, as well as to the act of grasping it, in reach-to-grasp arm movements (Tunik et al. 2005), and in the creation of cross-modal sensorial representations of objects (Grefkes et al. 2004). Therefore, the combination of findings from imaging studies on thalamo-cortical connectivity and from ex vivo experiments suggests the existence of a physiological connection between the cerebellum and the parietal cortex through the posterior VL thalamic nucleus. Our findings are in agreement with this hypothesis because the cerebello-thalamo-cortical pathway that we reconstructed mainly connects the cerebellum with the VA and VL thalamic nuclei. Further evidence of the coherence between our results and the literature is represented by the scarce connection we observed between the cerebellum and the parietal cortex through the posterior VL thalamic nucleus. Moreover, indications from literature suggest functional connectivity between the cerebellum and the parietal cortex (Buckner et al. 2011) but the existence of a direct anatomical pathway is still debated (Clower et al. 2005).

The temporal lobe encompassed 35 % of the tract-connected GM. Although the exact nature of connections between the temporal lobe (including the hippocampus and amygdala) and the cerebellum is still unclear, this connectivity is in line with studies showing that the temporal cortex indeed contributes to the cortico-pontine pathway both in humans and in macaque monkeys (Ramnani 2006). Indeed fMRI resting state (He et al. 2004) and dynamic causal modelling (Booth et al. 2007) have revealed functional connectivity between cerebellum and temporal areas, although this may in part depend on connections emitted by the fastigial nuclei through the middle cerebellar peduncle (at least in monkeys and cats) (Heath and Harper 1974).

Of the functional parcellation results (Table 2), the most striking finding was that the hemispheres of the cerebellum and the cortical associative areas encompassed 79 and 80 % of the tract GM, respectively. The associative cortex comprises the prefrontal (BA 9–12, 25, 46–47), parietal (except BA 1–3) and temporal (except BA 41–42) cortices and the limbic lobe. Since prefrontal, limbic and parts of parietal and temporal cortices are known to be involved in cognitive processes at different level of complexity (D’Angelo and Casali 2013), our results supports the theory that lateral areas of the cerebellum are also involved in higher cognitive processes (Schmahmann et al. 1999; Strick et al. 2009; Diedrichsen et al. 2009; Habas et al. 2009; Krienen and Buckner 2009; Watson et al. 2014).

The primary auditory and visual cortices only constituted 1 and 3 % of the tract-connected GM (Table 2), respectively, in line with the results from Buckner et al. (2011) who, using fMRI, have shown that primary auditory and visual cortices did not appear functionally connected with the cerebellum. On the other hand, these streamlines may be underestimated in this tractography study due to their relative position with respect to the cerebellum. Indeed, fibres connecting the cerebellum with visual and auditory areas (located in the occipital and temporal lobes, respectively) might have high curvature and therefore be partially undetected by tractography methods (e.g. see discussion in Buckner et al. 2011). A fairly recent DTI study (Doron et al. 2010) suggests that the cerebellum is strongly connected with the precentral gyrus and the superior frontal gyrus, which take part in motor and oculomotor processes as well as in the processing of spatial working memory (Du Boisgueheneuc et al. 2006). However, the very important role played by the cerebellum in controlling the execution of saccades, in elaborating the visuospatial information concerning the eye target (Tilikete et al. 2006; Guerrasio et al. 2010) and in controlling vestibulo-ocular reflexes, depends on connections emitted by the fastigial and vestibular nuclei through the inferior and middle cerebellar peduncles, which cannot be detected by placing a seed in the SCP.

An additional observation is the presence of a conspicuous number of streamlines connecting the cerebellum to the basal ganglia via the RN and the thalamus (Middleton and Strick 2002). Although the anatomo-functional relationship between basal ganglia and cerebellum remains unclear, a fast synaptic connection has recently been reported between these two structures (Chen and Khodakhah 2012), which also show coherent activity in fMRI recording (Mastropasqua et al. 2013). Moreover, basal ganglia are secondarily affected by atrophy in the presence of cerebellar damage (Dayan et al. 2013; Olivito et al. 2013). It has been postulated that a functional relationship between basal ganglia and cerebellum could be important for controlling movement (Amaral 2000). However, although our observation is in line with this concept, we have to point out that the present technique cannot be used to determine either the direction of the streamlines (projecting to or from the thalamus) or whether there are effective synaptic connections allowing communication between cerebellum and basal ganglia through the thalamus. Therefore, the nature of observed streamlines apparently connecting cerebellum and basal ganglia remains to be clarified.

Finally, our analysis also revealed streamlines reaching the septum. Again, although connections between the cerebellum and deep parts of the limbic system (including the septum) have been suggested (Heath et al. 1978), the synaptic nature and directionality of this pathway as well as functional evidence in humans await experimental confirmation.

### Limitations of the present study

While tractography is compelling in being applicable in vivo non-invasively, and hence in human subjects, it suffers from well-documented shortcomings. Although these have already been reported in several tractography publications, these limitations are discussed here in the context of the specific pathway under investigation in this study.

First of all, MRI tractography cannot distinguish between efferent and afferent fibres, since water diffuses equally in both anterograde and retrograde directions. The present results therefore cannot be used to inform models that rely on the determination of the direction of axon potential propagation.

Second, tractography methods cannot at present discriminate between direct and indirect connections between regions, since the diffusion weighted signal is influenced by the average microstructural architecture over the scale of an imaging voxel, and not by the directionality of the signalling process or the presence of synapses. Indeed, the cerebello-thalamo-cortical pathways are known to be polysynaptic and are not expected to form a direct connection between the cerebral and cerebellar cortices. In particular, it must be acknowledged that the connections to/from the VA and VL nuclei of the thalamus are complex, including not only fibres from the SCP but potentially also fibres from the basal ganglia. However, while tractography cannot identify regions of synapses, it is a mathematical algorithm with predefined rules and as such it may nonetheless be able to delineate onward connections if these rules are respected; for example, fibre-tracking algorithms typically require a certain degree of alignment between the fibre orientations estimated in neighbouring voxels; provided this requirement is satisfied, the algorithm will proceed through a region of synapses. If on the other hand the fitting of the fibre orientation distribution is noisy or does not capture the correct microstructure, the tractography algorithm could terminate even if there is continuity of the underlying biological tract.

One further limitation of tractography studies in general is that diffusion MRI data are rarely acquired at resolutions higher than 2 mm isotropic; this low spatial resolution is a considerable limitation when reconstructing pathways that converge onto a small structure and subsequently diverge towards a wider area of the brain. Here, we used a combination of CSD and super-resolution track-density imaging at 1 mm resolution to minimize this problem.

Another issue is related to the fact that tractography algorithms preferentially choose streamlines with minimal bending and there is a dependency of tract volume on path length and the tractography algorithm itself. Connections from the VA thalamic nucleus towards the prefrontal cortex have the highest trGM_cROI_ and TSC and also are characterized by minimal bending. Moreover, the anterior lobule of the cerebellum has the highest value of TSC and it is also the closest cerebellar region to the SCP seed point of the tract. However, the observed anatomical difference among these areas matches the expected difference in functional connectivity (Habas et al. 2009; Krienen and Buckner 2009), suggesting that, for this specific application, streamline connectivity revealed by our technique is not critically affected by anatomical constraints.

A final consideration on the validation of tractography results (Mori and van Zijl 2002) is that while tractography can indeed provide macroscopic neuroanatomical information on white matter pathways by reconstructing fibre structures that contain bundles of axons running along the same orientation, it cannot distinguish individual axonal pathways, whose diameter is typically less than 10 μm. For this reason, tractography cannot claim that the reconstructed tracts are anatomically accurate, and in fact results should be validated using other techniques. The most common way to infer information about axonal connectivity is using virus retrograde transport and chemical tract-tracing techniques in animals (Middleton and Strick 2002; Kelly and Strick 2003; Clower et al. 2005). The principal issue is that these techniques provide information at cellular level that cannot be compared directly to MR-derived results. Moreover, tract-tracing techniques cannot be applied to humans, where most information has come from post-mortem histological data (McNab et al. 2009; Miller et al. 2011; Seehaus et al. 2013; Dell’Acqua et al. 2013). An approach combining post-mortem dissection with advanced tractography seems best suited to characterize white matter architecture in humans and validate tractography results (Catani et al. 2012; Dell’Acqua et al. 2013), but requires the use of non-conventional scanners. A further way to validate tractography results is to compare the core of major white matter tracts with classical anatomical knowledge, because trajectories and locations of these tracts are fairly well known. However, the subcortical portions of the reconstructed tracts remain problematic due to the high uncertainty of fibre direction at the grey/white matter border.

Recent developments in tracking methods (e.g. Smith et al. 2012, 2013) may help minimize some of these effects in future work, and thus provide a more accurate estimate of the connections between cerebellar and cerebral cortices. Nonetheless, most of these limitations are inherent to diffusion MRI and will invariably need to be taken into consideration when interpreting any tractography results.

## Conclusions

We have shown that our advanced imaging methods allow visualization of the pathway connecting the cerebellar hemispheres with the contralateral cerebral cortex, passing through the SCP, red nucleus and VL and VA nuclei of the thalamus. The demonstration of congruent trGM_cROI_ of the cerebral and cerebellar cortices in functionally corresponding areas bears relevant functional implications. First, this result supports the coevolution of the two structures proposed on the basis of comparative cortical surface measurement across vertebrates (Sultan 2002). Secondly, since the cerebellar network has almost identical structure in all its sections and is organized in parallel poorly interacting modules (Standring 2008), it is possible that a similar computational cerebellar algorithm is applied to different cortical functions, ranging from motor control to sensory perception and cognition. This observation has special relevance for the generation of computational schemes and models of cerebro-cerebellar network loops (Ito 2008). Given that our advanced imaging analysis was successful using high-quality data acquired on standard clinical scanners, this method has immediate potential in the assessment of cerebellar structural connectivity in neurological conditions, for example in dyslexia and autism (e.g. Bauman and Kemper 2005; Boso et al. 2010) for which a cerebellar origin has been proposed (for review see D’Angelo and Casali 2013).

## Electronic supplementary material

Below is the link to the electronic supplementary material.

Supplementary material 1 (DOCX 2754 kb)
