# Supplementary material for: Contralateral cerebello-thalamo-cortical pathways with prominent involvement of associative areas in humans in vivo
Source: Brain Struct Funct. 2014 Aug 19;220(6):3369–84. doi: 10.1007/s00429-014-0861-2 (PMC4575696; doi:10.1007/s00429-014-0861-2)
Supplement: Supplementary file 1 — Supplementary material 1 (DOCX 2754 kb) [file 429_2014_861_MOESM1_ESM.docx]

**Supplementary Material**


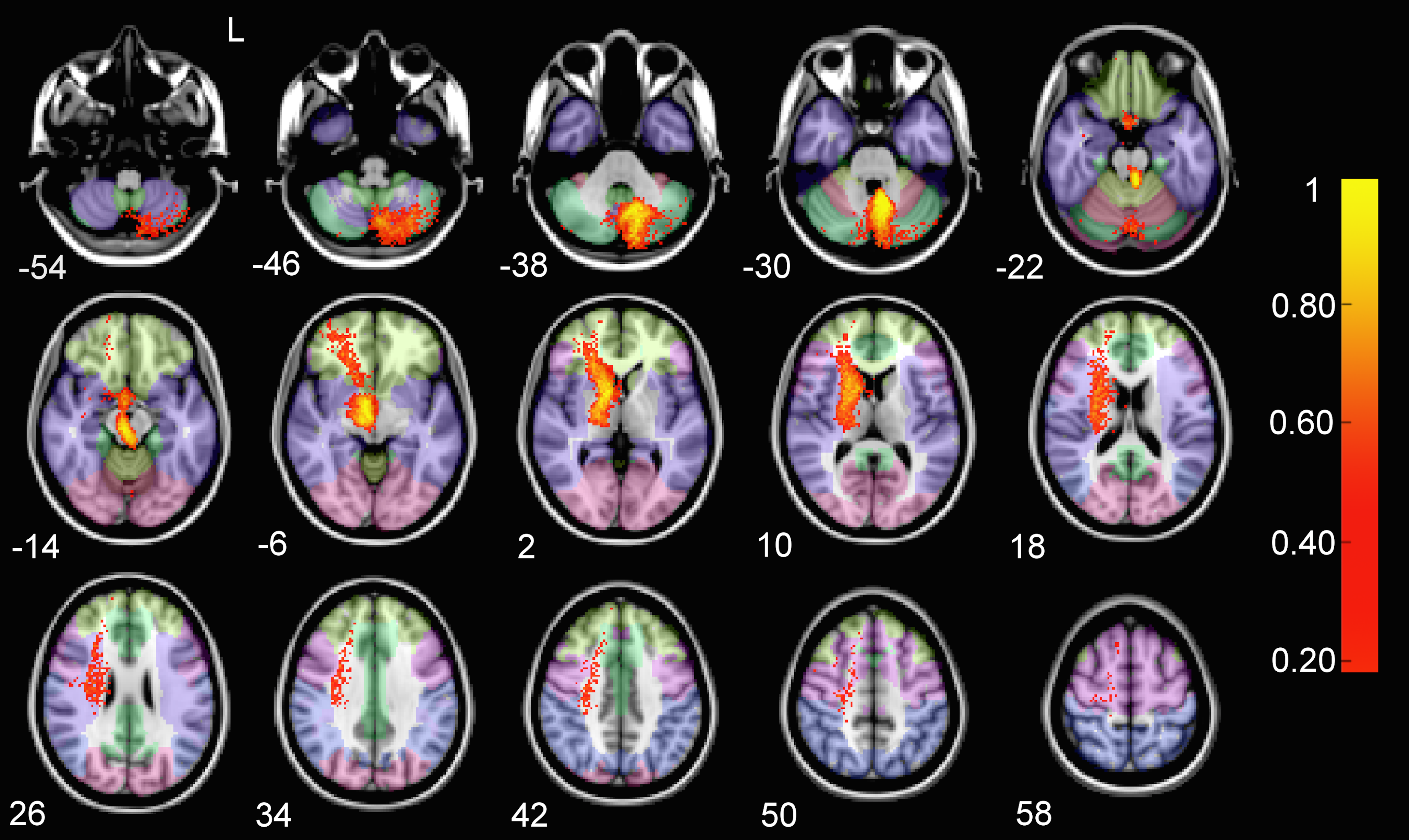


**Figure 1:** Extension of the average cerebello-thalamo-cortical pathway in whole brain from cerebellum to cerebral cortex in MNI space. Note fibre crossing below the thalamus (-14 mm) and prominent fibre density in the prefrontal cortex. For clarity, we have chosen to show only the left cerebello-cortical tract. Numbers correspond to the z coordinate (in mm) of each axial slice in MNI space. L indicates the left side of the brain. The scale on the right represents the legend of the mean pathway colour in terms of percentage of subjects.
